# The Details of Constructing a Porcelain Bridge

**Published:** 1900-04

**Authors:** G. W. Schwartz

**Affiliations:** Chicago.


ARTICLE V.

### THE DETAILS OF CONSTRUCTING A PORCELAIN BRIDGE.

G. W. SCHWARTZ, M. D., D. D. S., CHICAGO.

It is intended here to make clear many of the points in constructing a porcelain bridge that have given porcelain workers much concern, causing many to abandon the work.

It is conceded by all dentists that porcelain crown work has won its way to the front rank of prosthetic dentistry. With little argument against it when debated in dental societies, porcelain bridge work is still a bone of contention whenever the subject is introduced.

I will take for illustration a right superior bridge from the cuspid to the second molar, inclusive. We will suppose the roots are properly filled, and everything is ready to begin the preparation of the roots to be used as the abutments of the bridge.

I usually begin first by making a platinum cap for the second molar. I make a platinum cap for such a case because a long porcelain bridge is more likely to fracture than a short one. In this case it reduces the length of porcelain one-fifth, thus lessening the chances of fracture. If it were a four-tooth bridge from a cuspid to a first molar, the patient's first molar easily seen by the casual observer, I would make an all porcelain crown for the first molar, cutting the root even with the gum, making an abutement in just the same manner as I would for the cuspid root.

In preparing tho root for the metal cap I first cut down the tooth as near the gum line as desirable. I usually reduce the length of the crown of the tooth to about one-fourth its original length with enamel cleavers I trim the sides of the remaining tooth structure sufficiently parallel so that I can fit a band that will not destroy the interdental space, nor irritate the gum buccally nor lingually. The second molar now being prepared, I am ready for the measurements (to cut the metal) to make the band.

I prepare this abutment first because I may want to pare the cuspid at another sitting; for æsthetic reasons the patient might be annoyed at being without a cuspid longer than is absolutely necessary.

I usually take the measure of the root with fine binding wire. I always cut my hand longer at the occlusal than at the cervical because we must contour the band to get the flare at this part. By soldering the band in this way, we can trim down and get a perfect crown shape on this part of the band. When the band is properly festooned and fitted, the occlusal end should be filed and should be fitted perfectly square together and soldered with thirty per cent. platinum solder. If this is not done it is better to cut the band a trifle longer and make a lap joint. I plan the joint on the mesial part of the tooth, as it is afterward covered with the saddle.

Having the band fitted on, fill it with either modeling composition or plaster. Have the patient bite correctly, then remove the band and bite. Carve the cusps directly to this bite. Make die and counter die from an impression in moldine, strike up two cusps of platinum and solder them together with platinum solder. File them down until they are the proper length to be soldered to the band. They should be filed and honed until they are smooth and fit the band end to end, making a close joint. After they are soldered together we have a platinum cap that is twice as thick on the cusps as it is on the band, and if it is all made of twenty-eight gauge platinum, it is heavy enough to stand any strain put upon it. All that remains is to polish it. If properly constructed, it will occlude exactly when fitted on.

Next, the cuspid root should be cut off square to the gum line, and the labial surface beneath the free margin of the gum should be beveled. All other sides should be parallel. This is beveled labially because the facing can be ground to cover the band, and when completed will look as though it were a natural cuspid coming out of the gum, thus avoiding detection. Before the enamel is trimmed off a measure should be taken and laid away until needed.

When the metal is cut for the band it should be cut a trifle short to make allowance for the bevel on the root. The band should be driven on and made to take the shape of this bevel. This can only be done by having the band the size of the smallest part of the beveled root. After I have my band fitted I file and hone it down until it is even with the root, then solder a piece of iridio-platinum on for the cap, all of which is twenty-nine standard gauge. Having enlarged the root canal for a good sized post, punch a hole in the cap with a plate punch and file square with a small square jeweler's file, until it is large enough to take the square post, which should be about sixteen gauge and of iridio-platinum. Next fit the two in proper position on the root, letting the post extend out of the cap half the length of the tooth. If the post has been tightly fitted, it will bring the cap along with it and can be easily soldered, but if it has been loosely fitted, it will need to be invested to be soldered.

The pin is left long because it will be needed to solder the bar and pins to later. The joint between the bar and the abutements must be well made or we cannot hope for any degree of success. This is where most of the failures occur in the metal work, the frame to sustain the porcelain.

Having the abutments finished, place them on the roots in the mouth. Take a wax bite; next a plaster impression with the abutements in place. If they do not come away in the impression, remove them and replace them properly in the impression. Fill these abutments nearly full with melted wax before running the cast in the impression. This is to facilitate their removal from the model when it will be required later in soldering the bar, saddle, etc. When I run the cast in the impression I always allow it to extend an inch or two at the heel, so that I may make a plaster articulator when I run my cast in the wax bite. By doing this we have the normal occlusion. I cannot use the crown or bridge articulator as satisfactorily as I can the articulator made in this way.

After your models are made in plaster, take an impression of the model with the abutements in place in moldine, run a fusible metal die and counter-die. This is to be used for swedging the saddle. which should be very narrow on the ridge; I should say ordinarily, a saddle one-sixteenth of an inch is wide enough. The saddle should extend well on the mesial surface of the molar cap and cover the bottom of the cuspid, passing each side of the square post.

I usually build on a little wax at each approximal space on the model before I take the impression in moldine to run my dies to swedge the saddle. This is one of the places where I have seen failures, and have tried to remedy it by relieving the pressure at this point, which is distal to the cuspid and mesial to the molar. I have had hypertrophied growths at these points, and believe they were caused by the pressure of the saddle on the gum. It was on this account I introduced a double bar bridge about two years ago, which many dentists have approved and are now using instead of the saddle bridge.

Having the saddle swedged ready to solder, carefully remove the abutments from the model and melt the wax out of them. Replace them on the model, tako impression of them fn place on the model in modeling composition. The cap and crown should come away easily in the impression. Run a soldering cast in this impression with the abutments in it, and solder the saddle in place on this cast. This should be done with twenty-five per cent. platinum solder. The saddle should be made of twenty-eight gauge platinum.

Having the saddle soldered and the abutments replaced on the original plaster model, grind in the facings to their proper position, waxing them in place. Varnish the buccal surface of the model with sandarach. Let it dry, and flow plaster over the buccal surface of the model and facings, including the adjoining plaster tooth on each side. Remove the wax from the pins and saddle, fit in an iridio-platinum bar under the pins and well against the facings. The part of the bar that comes in contact with the molar cap should be flattened out so that a broad surface will come in contact with its lingual portion. This is to get a good strong point, as this is the point where I have seen most of the failures in porcelain bridge work.

Sixteen to eighteen gauge is the size bar or wire to use. The metal work must not be too light, as it will bend if it is, nor too heavy, as the porcelain will be liable to break away in this case.

Having the bar properly fitted in place, tack it to the cap and post of the cuspid abutment and molar cap with sticky wax. Now remove the teeth from the model, having them in the plaster investment. Put them aside for the present. Remove the metal work from the model and invest it through the center with a soldering investment. Remove the sticky wax solder, solder each end of the abutment to place. Remove the investment and replace the metal work on the model. Grind the pins in the facings flat where they come in contact with the bar. This is to give contact surface for the solder. If a round wire is used for the bar, flatten it also at this point for contact of the pins. Replace the facings in the investment back on the model. Wax the facings in place with sticky wax again and cut away the plaster investment from the teeth. The teeth will occupy the same position they were set in at the beginning. Varnish the buccal surfaces of the facings with sandarach. This is to prevent the investment burning on the teeth. They will come out of the investment clean, if this is done. Invest them, remove the wax without boiling.

Before soldering the pins to the bar, a stay should be put in between the bar and saddle midway between the cuspid cap and the molar cap. A wedge should be put in each end, one at the distal of the cuspid, and one at the medial of the molar, just between the saddle and the bar. This should all be soldered with twenty or twenty-five per cent. platinum solder. This makes it sure that the teeth will not change position going through the furnace. This done, the metal work is completed. The case is ready to receive the porcelain.

To be able to bake a porcelain bridge, one must know the anatomy of the natural teeth and how to carve. This can be accomplished only by careful study and much practice. Not many instruments are required, but a knowledge of their use is essential. I use a small spatula and an instrument for tracing the cusps, a medium sized screw pin vice, a small keyhole saw, two artists' brushes, No. 3 and No. 0.

Put the post of the cuspid in the screw pin vice. This makes a convenient way of handling the piece without danger of dropping it or getting the carving out of shape in handling. Begin by building up the molar first, slightly exaggerating the size, making allowance for shrinkage, next the second bicuspid and so on until it is completed and ready for the furnace.

To get the porcelain properly baked, when carving, from time to time, the saw should be drawn across the pin while holding the case. This brings the moisture to the surface much quicker by tapping. The moisture can readily be absorbed by an old clean piece of linen or absorbent paper. The work should be jarred down as much as possible before baking as this reduces the shrinkage to the smallest degree and insures better porcelain. After the piece is carved, it may be put on the model and tried for articulation, which should be a trifle full and allowed to shrink in the furnace. After the first bake, the piece will look different from the form first fashioned, being full of cracks and much shrunken. These should be filled up with new body and baked again, which is usually sufficient if no gum enamel is used. If the gum enamel is to be used, this should be done at a third baking. —*Items of Interest.*

